# TRSP is dispensable for the *Plasmodium* pre-erythrocytic phase

**DOI:** 10.1038/s41598-018-33398-8

**Published:** 2018-10-10

**Authors:** David Mendes Costa, Mónica Sá, Ana Rafaela Teixeira, Inês Loureiro, Catherine Thouvenot, Sylvain Golba, Rogerio Amino, Joana Tavares

**Affiliations:** 1 i3S – Instituto de Investigação e Inovação em Saúde, Universidade do Porto, Porto, 4200-135 Portugal; 2 IBMC – Instituto de Biologia Molecular e Celular, Universidade do Porto, Porto, 4200-135 Portugal; 3 Center for Production and Infection of Anopheles, Institut Pasteur, Paris, 75015 France; 4 Unit of Malaria Infection and Immunity, Institut Pasteur, Paris, 75015 France; 5 Present Address: Ultrapole, Institut Pasteur, Paris, 75015 France

## Abstract

*Plasmodium* sporozoites deposited in the skin following a mosquito bite must migrate and invade blood vessels to complete their development in the liver. Once in the bloodstream, sporozoites arrest in the liver sinusoids, but the molecular determinants that mediate this specific homing are not yet genetically defined. Here we investigate the involvement of the thrombospondin-related sporozoite protein (TRSP) in this process using knockout *Plasmodium berghei* parasites and *in vivo* bioluminescence imaging in mice. Resorting to a homing assay, *trsp* knockout sporozoites were found to arrest in the liver similar to control parasites. Moreover, we found no defects in the establishment of infection in mice following inoculation of *trsp* knockout sporozoites via intravenous and cutaneous injection or mosquito bite. Accordingly, mutant sporozoites were also able to successfully invade hepatocytes *in vitro*. Altogether, these results suggest TRSP may have a redundant role in the completion of the pre-erythrocytic phase of the malaria parasite. Nonetheless, identifying molecules with paramount roles in this phase could aid in the search for new antigens needed for the design of a protective vaccine against malaria.

## Introduction

Malaria is a vector-borne infectious disease caused by apicomplexan parasites belonging to the genus *Plasmodium*. It remains one of the most concerning public health problems with 216 million cases reported in 2016 leading to nearly half a million deaths, most of which were children under the age of 5 in Sub-Saharan Africa^1^.

A considerable effort has been made to generate a vaccine against malaria. In fact, several projects are currently underway^2^ and, most notably, the circumsporozoite protein (CSP)-targeting RTS,S/AS01 has completed Phase 3 clinical trials^3^. Nonetheless, the protection conferred by RTS,S/AS01 against malaria in young children is only partial and, therefore, more effective options are required.

The pre-erythrocytic phase of malaria is initiated when an infected female anopheline mosquito injects sporozoites in the skin of a mammalian host while taking a blood meal. Only a few sporozoites eventually leave the skin and travel through the bloodstream to reach the liver. Inside hepatocytes they differentiate and multiply into merozoites, the red blood cell-infective forms. These are subsequently released into the bloodstream to initiate the symptomatic erythrocytic phase^4^.

Prior to hepatocyte invasion, sporozoites passively transported in the blood must arrest in the liver sinusoids, a crucial step for the ensuing liver infection. This arrest has been proposed to be enabled by both slower blood circulation speed in the sinusoids and the interaction of sporozoite surface molecules such as CSP and the thrombospondin-related anonymous protein (TRAP) with the highly-sulphated heparan sulphate proteoglycans (HSPGs) of liver cells^5–8^. It is noteworthy that the current model explaining this critical step is based on the interpretation of experiments where the binding of recombinant proteins to hepatocytes was tested or the outcome of a liver infection by sporozoites was assessed. Using *in vivo* bioluminescence imaging, we have recently demonstrated that *Plasmodium* sporozoites home to the liver in the first minutes following their intravascular inoculation in mice^9^. Thus, the identification of the parasite determinants involved in this stage-specific behaviour could be possible by generating transgenic parasites lacking candidate surface molecules and following their capacity to home to the liver by direct imaging *in vivo*.

The thrombospondin-related sporozoite protein (TRSP), a protein found on the parasite surface and previously implicated in the pre-erythrocytic phase, is among those candidates^10,11^. TRSP possesses an adhesive thrombospondin type 1 repeat (TSR) domain that is conserved throughout *Plasmodium* species and shows great similarity to those of TRAP and CSP^10,12^. TSRs are present in a diverse group of proteins, promote cell-cell and cell-matrix interactions in a wide array of organisms, and can bind to proteoglycans^13–15^. It has been proposed that TRAP and CSP TSRs bind specifically to HSPGs in the liver^5,7,16,17^. In the absence of TRSP, *Plasmodium berghei* sporozoites have been shown to present compromised hepatocyte entry *in vitro*, which correlated with lower parasite burden in the liver^10^. However, the role of this protein in mediating the arrest of sporozoites in the liver, a step that precedes hepatocyte infection, has never been addressed.

In this work, we have engineered luciferase-expressing *P*. *berghei* sporozoites lacking TRSP and evaluated their capacity to home to the liver using bioluminescence imaging. Moreover, we found no defects in the infectivity of *trsp* knockout sporozoites suggesting this molecule has a redundant role in the pre-erythrocytic phase of malaria.

## Results

### *trsp* knockout sporozoites efficiently target the liver

In order to investigate the potential role of TRSP in the homing of salivary gland sporozoites to the liver, we resorted to a homing assay using live imaging^9^. Briefly, making use of 2D bioluminescence imaging in mice it is possible to quantify the specific accumulation of luciferase-expressing sporozoites in the liver in the first minutes following their intravascular inoculation^9^. Thus, we generated a *trsp* knockout line on a bioluminescent background via targeted gene deletion by double crossover homologous recombination (Supplementary Fig. 1A). Transfer populations were cloned to select isogenic lines hereby designated TRSP^-^. The two clonal parasite lines used throughout this work, TRSP^-^ I21 and I26, were confirmed to have integrated the selectable marker to replace the *trsp* locus via PCR (Supplementary Fig. 1B) and Southern blot (Supplementary Fig. 1C), and shown to lack *trsp* mRNA by RT-PCR (Supplementary Fig. 1D).

**Figure 1:**
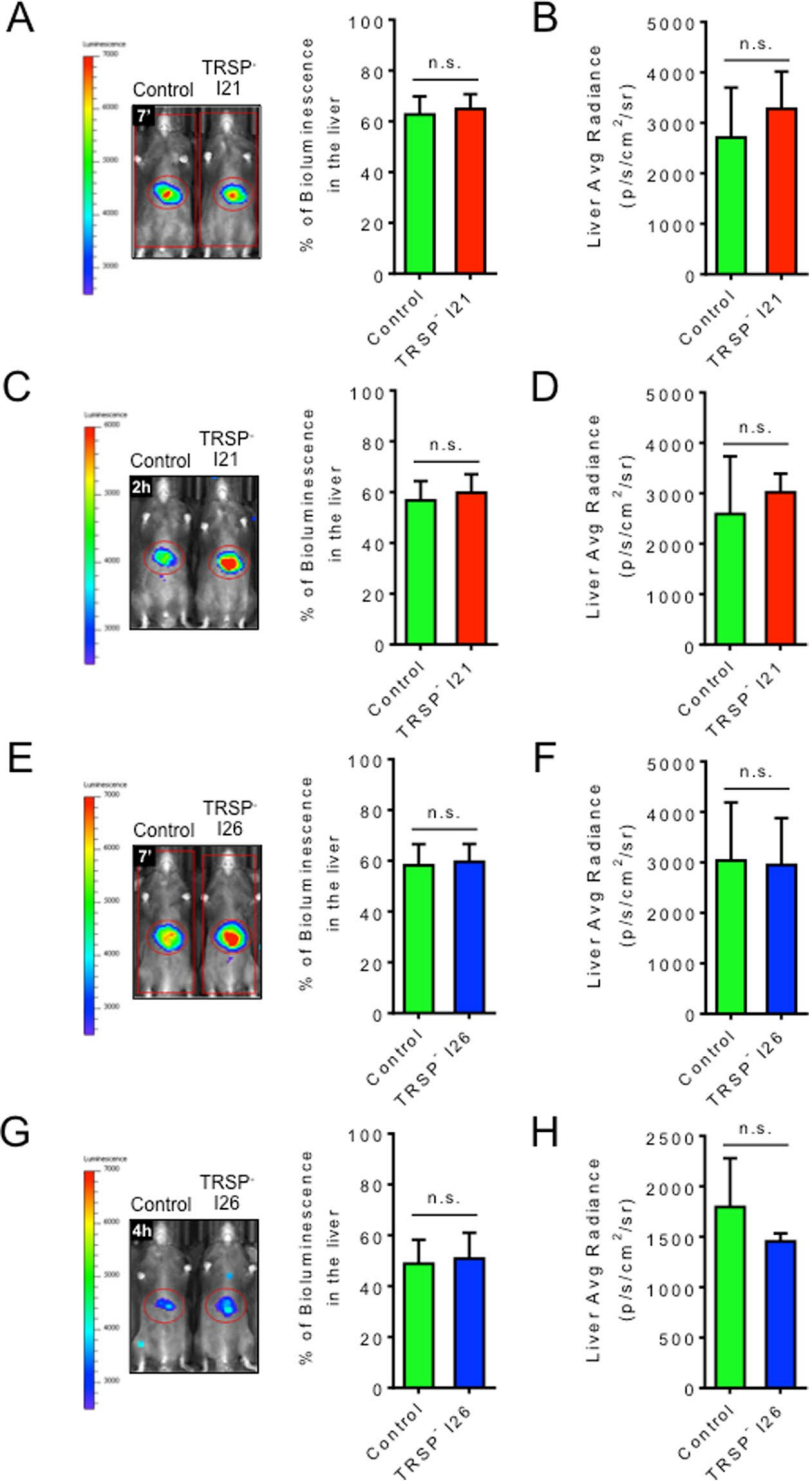
Homing of *trsp* knockout sporozoites to the liver. (**A**,**C**,**E**,**G**) Representative pictures of C57BL/6 mice injected intravenously with 1.5 × 10^5^ luciferase-expressing control or TRSP^-^ I21 (**A**,**C**) or I26 (**E**,**G**) sporozoites collected from mosquito salivary glands. Quantification of the signal in percentage of total flux (photons/s) in the liver versus total body signal (mean ± SD; n = 3), determined 7 minutes (**A**,**E**) or 2–4 hours (**C**,**G**) after sporozoite injection. (**B**,**D**,**F**,**H**) Bioluminescent signal in the liver plotted as average radiance (photons/s/cm^2^/sr; mean ± SD; n = 3) of the mice infected with TRSP^-^ I21 (**B**,**D**) or I26 (**F**,**H**) sporozoites 7 minutes (**B**,**F**) or 2–4 hours (**D**,**H**) post-infection. Data shown is representative of two independent experiments. Statistical analysis was performed using the Mann Whitney test: n.s., non-significant.

In the homing assay, we detected no difference in the bioluminescent signal in the liver 7 minutes after mice being inoculated with control versus TRSP^-^ sporozoites either in percentage (Fig. 1A,E) or absolute values (Fig. 1B,F). This indicates that sporozoites lacking TRSP efficiently home to the liver. In addition to the short 7-minute time point, bioluminescence was also quantified 2 to 4 hours post-infection and also no significant differences in the bioluminescent signal either in percentage (Fig. 1C,G) or absolute values (Fig. 1D,H) among the different groups were observed. Altogether, these observations demonstrate that *trsp* knockout parasites are able to successfully reach the liver.

### TRSP is dispensable for the completion of the *P*. *berghei* pre-erythrocytic phase

**Figure 2:**
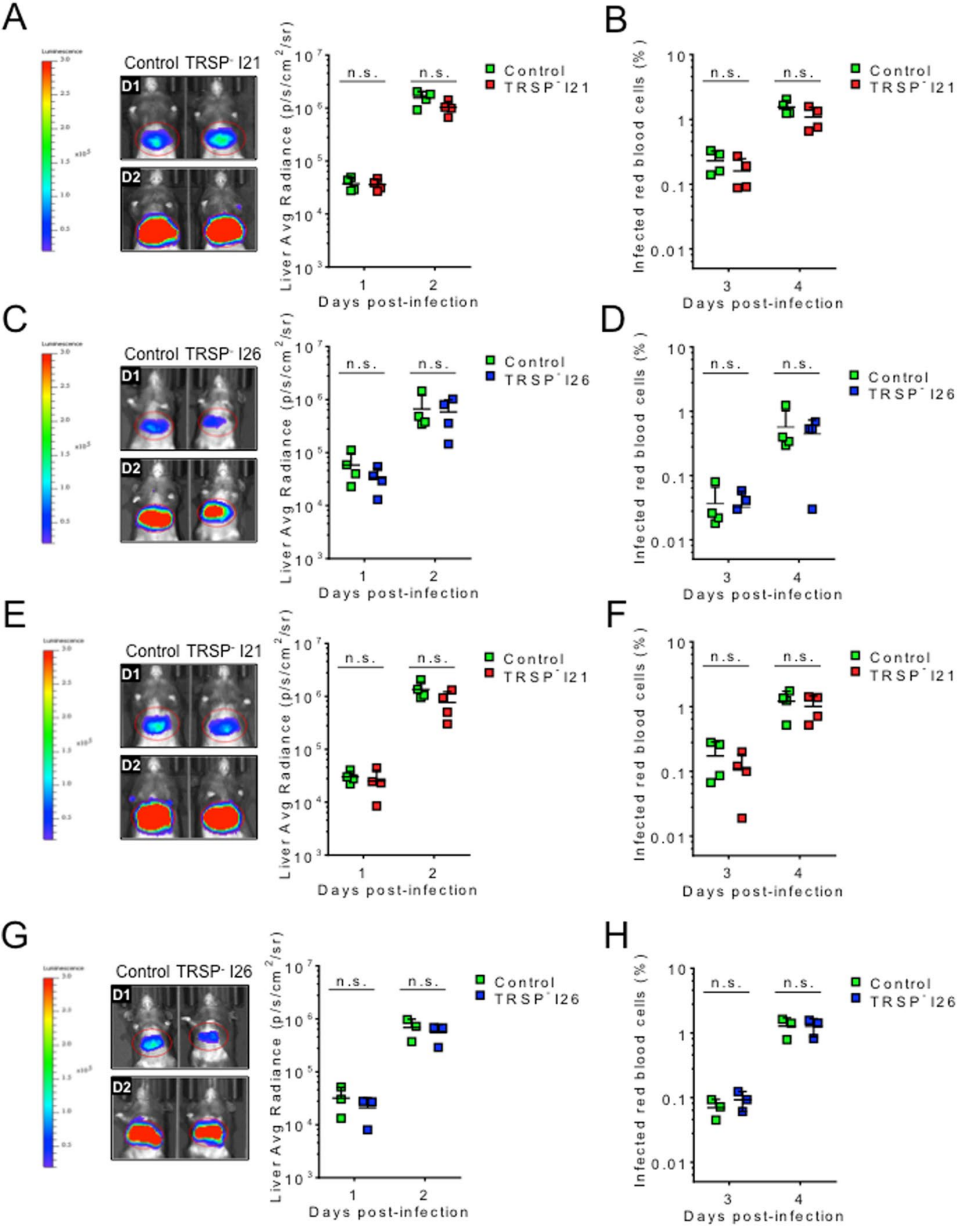
Infectivity of *trsp* knockout sporozoites in the mammalian host following intravenous or intradermal infection. Infectivity of TRSP^-^ (clones I21 or I26) and control sporozoites following infection by intravenous (**A**–**D**) or intradermal (**E**–**H**) routes. In these representative experiments, intravenous infections were carried out using 1 × 10^4^ sporozoites, while intradermal inoculations were performed with 1.5–2 × 10^4^ sporozoites. Parasite burden in the liver (n = 3 or 4) was assessed by live imaging on days 1 (D1) and 2 (D2) post-infection using IVIS Lumina LT. The bioluminescent signal as average radiance (photons/s/cm^2^/sr) of individual mice (symbols) and the average +SD (bars) of groups were plotted (**A**,**C**,**E**,**G**). Starting on day 3 post-infection, parasitaemia of infected animals was determined daily by a Giemsa-stained blood smear. Parasitaemia of individual mice (symbols) and the average +SD (bars) of groups are represented (**B**,**D**,**F**,**H**). Statistical analysis was performed using the Mann Whitney test: n.s., non-significant.

To evaluate the capacity of intravenously-injected sporozoites to infect hepatocytes, bioluminescence in the liver was quantified at days 1 and 2 post-infection (Fig. 2A,C) and the percentage of infected red blood cells was calculated on the following days (Fig. 2B,D). No differences in bioluminescent signal in the liver or in the percentage of infected red blood cells were observed between mutant and control parasites. Since intravenous inoculation bypasses the critical skin stage of the sporozoite journey to the mammalian liver, mice were also infected intradermally (Fig. 2E–H). Similarly, TRSP^-^ parasites did not show any defects in the liver (Fig. 2E,G) and blood (Fig. 2F,H) infection. In conclusion, loss of TRSP does not impact the ability of sporozoites to reach and infect the liver, regardless of the infection route.

**Figure 3:**
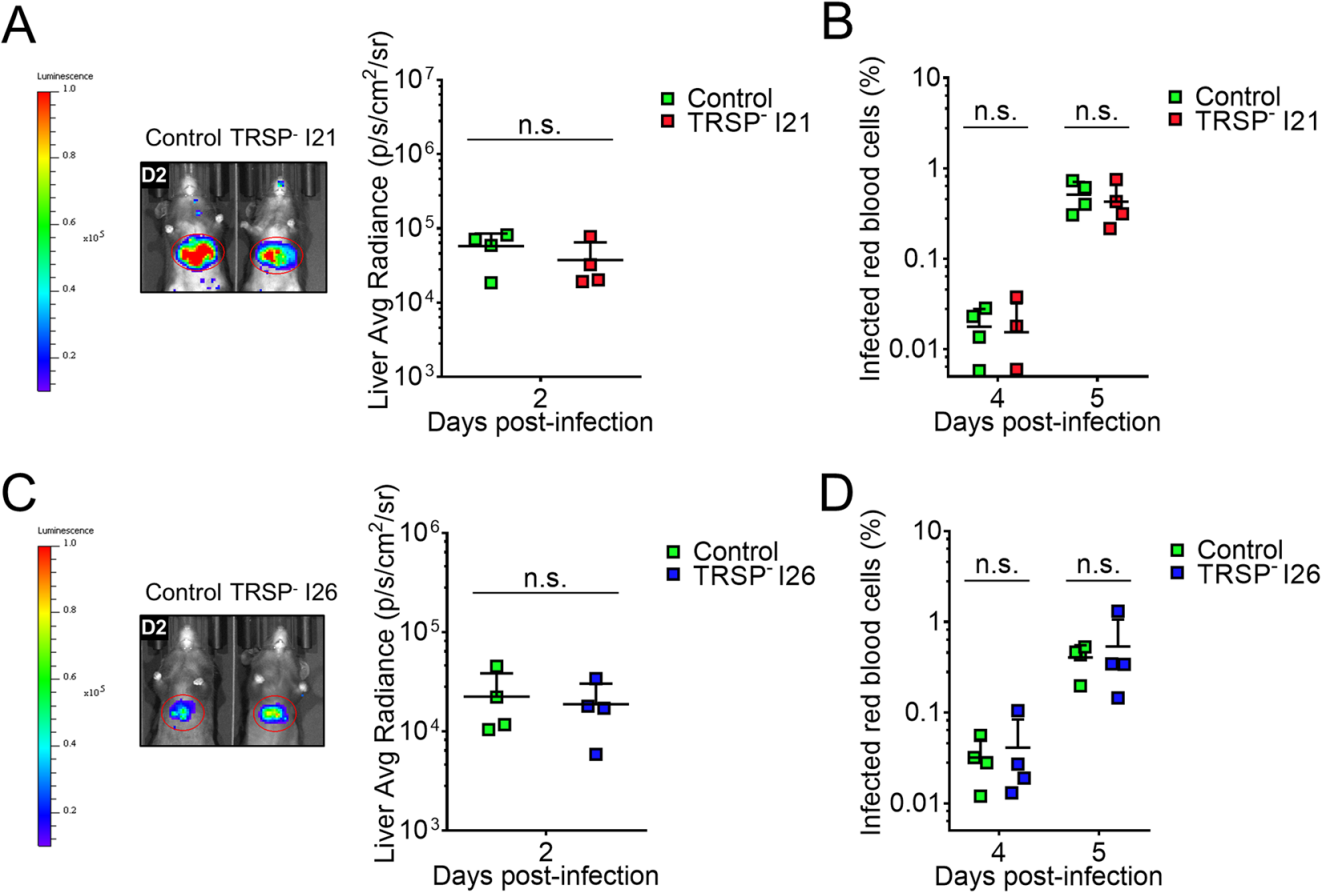
Infectivity of TRSP^-^ sporozoites through mosquito bite. Mosquitoes infected with TRSP^-^ I21 (**A**,**B**) or I26 (**C**,**D**) and control parasites were used in bite experiments. Five to six mosquitos/mouse were allowed to probe for a total of 25 consecutive minutes. Parasite burden in the liver was assessed by live imaging on day 2 (D2) post-infection using IVIS Lumina LT. Bioluminescent signal was quantified and plotted as average radiance (photons/s/cm^2^/sr) (**A**,**C**). Starting on day 3 post-infection, parasitaemia of infected animals was determined daily by a Giemsa-stained blood smear (**B**,**D**). Values for individual mice (symbols) and the average +SD (bars) of groups are represented. Statistical analysis was performed using the Mann Whitney test: n.s., non-significant.

Although intravenous and intradermal infections are helpful experiments to scrutinize the behaviour of the parasites in specific stages of the pre-erythrocytic phase, these circumvent the deposition of sporozoites and mosquito saliva into the skin through the mosquito proboscis. To study the importance of TRSP in the context of natural transmission, infected mosquitos were allowed to probe on naive mice and the bioluminescent signal in the liver was measured two days after infection (Fig. 3A,C). Data obtained on day 1 were not considered because infections following mosquito bite typically lead to low parasite burdens in the liver when compared to intravenous (Fig. 2A,C) or intradermal (Fig. 2E,G) infections at this time point. No impairment of liver infection was discernible on day 2 (Fig. 3A,C). Inspection of parasitaemia levels on later days also did not reveal differences (Fig. 3B,D).

Overall, these data demonstrate that TRSP^-^ are as capable as control sporozoites of accomplishing the pre-erythrocytic phase of their life cycle. Furthermore, mutant parasite growth during the blood stage was indistinguishable from that of control parasites (Figs 2B,D,F,H and 3B,D). In addition, comparable numbers of salivary gland sporozoites were observed (Supplementary Fig. 2), revealing that no problems in transmission to or infection of the mosquito occurred. This is further evidence that TRSP is not necessary for the *P*. *berghei* life cycle in standard conditions.

### TRSP^-^ sporozoites exhibit no impairment in hepatocyte entry

**Figure 4:**
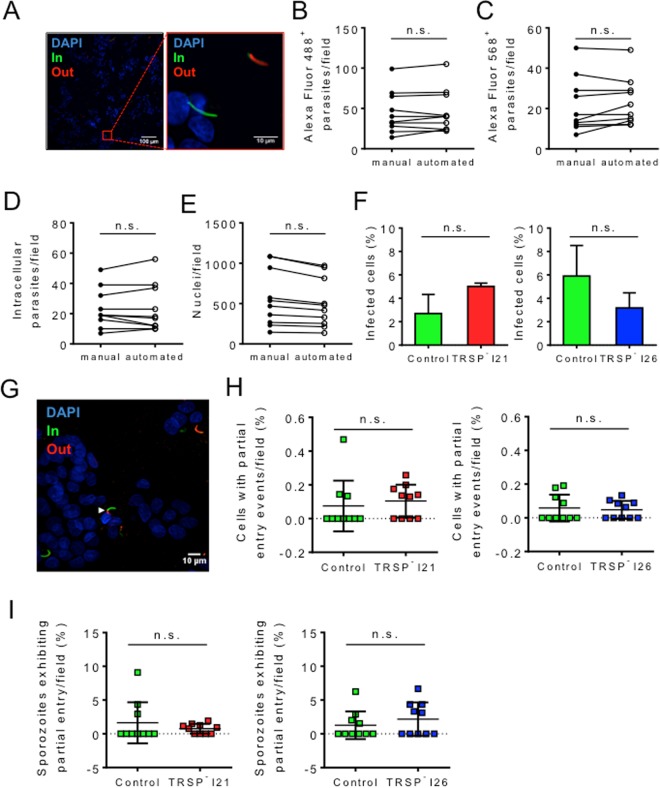
TRSP^-^ sporozoite entry in hepatocytes. (**A**) Representative immunofluorescence image of an anti-CSP double staining using control sporozoites in an *in vitro* entry assay. Intracellular sporozoites (In) are labelled in green whilst extracellular sporozoites (Out) appear in red and green. DAPI-stained nuclei appear in blue. The image on the right represents a magnified section of the image on the left. (**B**–**E**) Comparison of the number of sporozoites per field stained with anti-CSP antibodies and Alexa Fluor 488-conjugated (**B**) or Alexa Fluor 568-conjugated (**C**) secondary antibodies; intracellular sporozoites (**D**), corresponding to the number of Alexa Fluor 568^+^ parasites subtracted from the number of Alexa Fluor 488^+^ parasites; and nuclei (**E**) in a representative anti-CSP double staining, determined manually or by automated analysis. (**F**) Percentage of HepG2 cells infected by control and TRSP^-^ I21 (graph on the left) or I26 (graph on the right) sporozoites, in 25 fields from each replicate well. (**G**) Partial entry events were defined as Alexa 488^+^ sporozoites stained with Alexa 568 secondary antibody up to roughly 50% of its length in an anti-CSP double staining. A partial entry event of control sporozoites in an immunofluorescence image is identified with a white arrowhead. DAPI-stained nuclei appear in blue (**H**) Percentage of cells exhibiting partial entry events using TRSP^-^ I21 (graph on the left) or I26 (graph on the right) sporozoites. Ten fields of a representative assay were analysed and events were counted manually. (**I**) Percentage of sporozoites partially inserted into the hepatocyte membrane using TRSP^-^ I21 (graph on the left) or I26 (graph on the right) sporozoites. This parameter represents the number of partial entry events out of the total number of intracellular parasites in a field, represented as a percentage. Means + SD (**F**) or means ± SD (**H,I**) are represented. Four independent experiments per clone were performed. Statistical analysis was performed using the Mann Whitney test: n.s., non-significant.

A particular phenotype relating to defective sporozoite entry into hepatocytes was previously described *in vitro* for *trsp* knockout parasites^10^. We inspected the ability of the TRSP^-^ clonal lines generated in this study to invade the HepG2 hepatoma cell line using an automated counting method to examine anti-CSP double staining, which distinguishes between intracellular and extracellular sporozoites^18^. Images were acquired using an IN Cell Analyzer 2000 and the numbers of host cell nuclei, total and intracellular sporozoites were counted either manually or using the IN Cell Developer Toolbox v.1.9.2 software (Fig. 4A–E). The two counting methods yielded indistinguishable results (Fig. 4B–E), validating the use of the automated method to determine the percentage of infected cells (Fig. 4F). No statistically significant differences were observed between TRSP^-^ and control sporozoites, indicating TRSP^-^ sporozoites have no major defects in host cell entry. These results also differ from the former study on the *P*. *berghei trsp* knockouts, where the authors described a failure in sporozoite entry into HepG2 cells that culminated in an incomplete entry phenotype^10^. We failed to observe this phenotype with our TRSP^-^ lines (Fig. 4G–I). In fact, the partial entry of sporozoites was an extremely rare event in both control and mutant parasites (Fig. 4G). At most, only approximately 0.1% of host cells exhibited such events using any of the parasite lines (Fig. 4H) and these events did not constitute more than 2.1% of total intracellular parasites (Fig. 4I) in the selected representative experiments.

## Discussion

This is the first study where the importance of a sporozoite surface protein in the homing of *Plasmodium* parasites to the liver was investigated using live imaging techniques. We generated TRSP^-^ bioluminescent clonal lines and made use of a homing assay to dissect the role of this protein in the specific retention of sporozoites in the liver. Strikingly, we found that TRSP not only is not required for the homing of sporozoites to the liver, but is also dispensable for the establishment of a successful infection in the mammalian host. These results challenge the findings of a previous study that advocated an involvement of TRSP in hepatocyte entry and *P*. *berghei* infectivity^10^. The paucity of the *in vitro* partial entry events could, to an extent, explain the disagreeing results. Nonetheless, the infrequency of incomplete hepatocyte entry events in our experiments suggests this phenomenon is most likely not biologically relevant. Moreover, different methods were used in both studies to evaluate the liver infectivity of the TRSP-deficient sporozoites, as in the previous study parasite burdens in the liver were assessed using RT-PCR^10^. We cannot exclude that the measurement of parasite burden using bioluminescence imaging is less sensitive than RT-PCR. However, no differences in the emergence of the blood infection following inoculation of TRSP^-^ or control sporozoites were found in our study, supporting a similar capacity for both parasites to infect the liver. Once again, this contrasts with the one-day delay in the pre-patent period reported in the former work^10^. Noteworthy, in this study we used the *P*. *berghei* ANKA strain rather than the NK65 strain^10^. Despite the high similarity between the genome sequences of several *P*. *berghei* laboratory lines^19^ we cannot preclude that such phenotypic discrepancies may come from the fact that the *trsp* knockouts were engineered in distinct strains.

The processes in which TRSP may participate remain therefore undisclosed. The *trsp* gene in *P*. *berghei* codifies for a relatively small 159 amino-acid protein with a signal peptide, a transmembrane domain and a short cytoplasmic tail, in addition to the adhesive TSR^10^. TRSP has been demonstrated to have apical localization in *Plasmodium yoelli*^12^ and has also been readily detected on the surface of purified and untreated *P*. *falciparum* and *P*. *yoelii* salivary gland sporozoites^11,20^. The TRSP exact subcellular localization and the mechanism that regulates its trafficking to the sporozoite surface remain to be elucidated. *trsp* transcripts are sporozoite-specific and are enriched in salivary gland sporozoites in comparison to their midgut-associated counterparts^12,21^. Hence, TRSP would be predicted to play a part in the initial stages of the *Plasmodium* life cycle in the mammalian host, in processes such as motility, adhesion or invasion^22,23^. On the other hand, the similarities shared between TRSP and other sporozoite surface proteins, namely those belonging to the TRAP family and other TSR-containing proteins, lead us to speculate that functional redundancy among these molecules may exist. This phenomenon has already been reported in similar contexts for other proteins^24,25^. It is possible that other adhesins compensate for the loss of TRSP and allow for the parasite to complete its life cycle without any major impairment.

Finally, the fact that TRSP appears to be dispensable for the parasite to successfully infect the mammalian host dampens the hope that this surface molecule could become an attractive target for neutralizing antibodies^11^. Nonetheless, the identification of sporozoite surface proteins with functions that are vital to the completion of the sporozoite journey in the mammalian host remains a promising approach to design a novel vaccine. Stimulating a humoral response against these antigens would not only directly target the parasites and facilitate their elimination but also block crucial steps of the life cycle and, subsequently, impede the natural progression of infection.

## Materials and Methods

### Ethics statement

All experiments carried out on mice were approved by the IBMC.INEB Animal Ethics Committees and the project was licenced by the Portuguese National Authority for Animal Health, in accordance to the statements on the directive 2010/63/EU of the European Parliament and Council.

### Mice, parasites and mosquitoes

Four- to six-week-old C57BL/6 or NMRI mice used in this study were purchased from Charles River or the IBMC/i3S animal facility.

*P*. *berghei* ANKA strain clone 676cl1 expressing a GFP-luciferase fusion gene via the pbef1 promoter (GFP:LUC)^26^ was used to generate mutant lines and was included in experiments the parental control.

*Anopheles stephensi* mosquitoes (Sda500 strain) were reared in the Centre for Production and Infection of Anopheles (CEPIA) at the Pasteur Institute using standard procedures. Female mosquitoes were fed on infected NMRI mice as previously described^27^ and from 21 to 28 days after the infectious blood meal these were either dissected for collection of salivary gland sporozoites or used in transmission experiments. In the latter scenario, starvation by sucrose deprivation 1 day prior to experimentation was performed to enhance the rate of mosquito bite.

### Generation of TRSP^-^ clonal lines

Targeted deletion of *trsp* by double crossover homologous recombination was achieved using a previously described strategy^10^, with modifications. To this end, the 3′ and 5′ UTR sequences used as homology regions were amplified from genomic DNA using a Taq DNA polymerase with proofreading activity (Takara) and primer pairs P1 + P2 and P3 + P4 (Supplementary Table 1), respectively. Amplicons were cloned into pGEM-T Easy Vector (Promega), sequenced and later subcloned into the KpnI/HindIII or EcoRI/BamHI sites of pL0001 vector (MR4; 5′ or 3′ homology regions, respectively). The linearized targeting vector resulting from KpnI/BamHI digestion was used to transfect schizonts with the Nucleofector® device (Amaxa)^28^. Briefly, electroporated merozoites were injected intravenously into two mice (parental populations) and selected by pyrimethamine treatment at 0.07 mg/L in drinking water, started the day following infection. Once parasitaemia was above 1%, blood from each animal was transferred to two naive mice (transfer populations) for a second round of selection. Parasites were then cloned by limiting dilution.

### Genetic validation by PCR and Southern blot analysis

#### Genomic DNA extraction

Blood collected from infected animals was filtered with a Plasmodipur leukocyte filter (EuroProxima), followed by erythrocyte lysis with saponin (0.15%). DNA was then extracted and purified from the resulting parasite pellet using the QIAamp DNA Blood Mini Kit (Qiagen).

#### PCR

Absence of the *trsp* open reading frame (ORF) and integration of the transfection construct at the correct locus were confirmed by standard PCR analysis with primer pairs P5 + P6 and P7 + P8 (Supplementary Table 1), respectively.

#### Southern blot

For Southern blot analysis, 5 µg of genomic DNA were digested with EcoRV, separated on a 0.8% agarose gel and transferred to a Hybond-N + nylon membrane (Amersham). The DNA sequence amplified by PCR using primer pair P1 + P2 (Supplementary Table 1) was used as a probe. Labelling of the probe and signal generation and detection were performed resorting to the AlkPhos Direct Labeling and Detection System with CDP-Star chemiluminescent detection reagent (Amersham).

### Evaluation of gene expression by reverse transcription PCR (RT-PCR)

Total RNA was isolated from salivary gland sporozoites using the Nucleospin RNA II kit (Macherey-Nagel) and reverse transcription for cDNA production was achieved with the NZY First-Strand cDNA Synthesis Kit (NZYtech). Primer pair P5 + P9 (Supplementary Table 1) was utilised to detect *trsp* cDNA by PCR. Tubulin beta chain gene cDNA was used as a control, employing primer pair P10 + P11 (Supplementary Table 1).

### Hepatocyte entry assays

#### Immunofluorescence assays

The assays were conducted by seeding 7.5 × 10^4^ to 1 × 10^5^ HepG2 cells (ATCC HB-8065) in an 8-well Lab-Tek chamber slide (Thermo Fisher Scientific) in duplicates or triplicates. Cells were cultured at 37 °C in DMEM 10% FBS, left to adhere overnight and infected with 1 × 10^4^–5 × 10^4^ sporozoites. Preparations were then fixed with 4% paraformaldehyde 1 hour post-infection.

The ability of sporozoites to enter HepG2 cells *in vitro* was evaluated via an anti-CSP double staining. Preparations were blocked with 5% FBS in PBS, incubated with an α-CSP 3D11 mouse monoclonal antibody (~2 µg/ml; MR4), and then with the goat α-mouse Alexa Fluor 568 antibody (1:500; Invitrogen). Permeabilization of the host cell membrane with 1% Triton 100 in PBS ensued, followed by incubation with the primary antibody. Finally, the goat α-mouse Alexa Fluor 488 (1:500; Invitrogen) antibody was used to stain all parasites. This allowed for the discrimination between intracellular (Alexa Fluor 488^+^/Alexa Fluor 568^−^) and extracellular (Alexa Fluor 488^+^/Alexa Fluor 568^+^) parasites. Nuclei were stained with DAPI (1:5000; Invitrogen).

#### Automated analysis

Image acquisition was performed using IN Cell Analyzer 2000 (GE Healthcare). HepG2 nuclei as well as green and red sporozoites were counted using the IN Cell Developer Toolbox v.1.9.2 software (GE Healthcare). In hepatocyte entry assays, 25 images corresponding to 0.57 mm^2^ fields were taken per well. Automated slide analysis was validated in all experiments by comparison of values obtained with the developed protocol and manual counts using ImageJ (National Institutes of Health) for randomly selected images.

### Mouse infections

For homing experiments^9^, 1.5 × 10^5^ sporozoites were injected intravenously in C57BL/6 mice. In the other experiments, C57BL/6 mice were infected with 1 × 10^4^–1.5 × 10^4^ or 1.5 × 10^4^–2 × 10^4^ sporozoites injected intravenously or intradermally, respectively. To assess the role of TRSP in the context of natural transmission, approximately 25–40 mosquitoes were allowed to probe mice anaesthetized with ketamine (125 mg/kg) and xylazine (12.5 mg/kg) in sessions of 1 minute with rotation of the animals, for a total of 25 consecutive minutes.

### Bioluminescence imaging

Liver parasite loads in C57BL/6 mice infected with luciferase-expressing sporozoites were assessed by bioluminescence imaging using the IVIS Lumina LT system (Perkin Elmer). Prior to image acquisition, animals had their ventral fur shaved with an appropriate clipper. Infected mice were anaesthetized with 2.5% isoflurane (O_2_ flow of 1 L/min) and injected subcutaneously with D-luciferin potassium salt (2.4 mg, Perkin Elmer) five minutes before image acquisition. Mice were then transferred to the stage of an intensified charge-coupled device photon-counting video camera box where anaesthesia was maintained with 2.5% isoflurane (O_2_ flow of 0.3 L/min). Signal acquisition was controlled by the Living Image software (Perkin Elmer). In the end, animals returned to their cage and recovered from the anaesthesia. The detection of bioluminescent signals by the system resulted in the generation of signal maps automatically superimposed to the grey-scale photograph of the mice. The quantifications were performed using the Living Image software (Perkin Elmer). The regions of interest (ROI) encompassing the liver and, in homing assays, the whole ventral views of the animal body were manually defined^9^. The total flux (photons/second) and average radiance (photons/second/cm^2^/steradian) within these ROIs were automatically calculated. In the homing assays, the percentage of bioluminescent signal in the liver ROI was calculated by dividing the total flux of the respective ROI by the total flux of the ROI of the animal body in the ventral position.

### Parasitaemia

The percentage of infected red blood cells was evaluated by Giemsa-stained thin blood smear starting from the third day after sporozoite inoculation. At least 40 microscopic fields (~ 20,000 red blood cells; detection limit 0.005%) were analysed.

### Statistical analysis

Mann-Whitney test was performed with GraphPad Prism Software (version 6.0). Statistical significance was found for values of p < 0.05.

## Electronic supplementary material

Supplementary Information
