# Supplementary material for: TRSP is dispensable for the Plasmodium pre-erythrocytic phase
Source: Sci Rep. 2018 Oct 10;8:15101. doi: 10.1038/s41598-018-33398-8 (PMC6180128; doi:10.1038/s41598-018-33398-8)
Supplement: Supplementary file 1 — Supplementary Information [file 41598_2018_33398_MOESM1_ESM.pdf]

TRSP is dispensable for the *Plasmodium* pre-erythrocytic phase

David Mendes Costa[1,2], Mónica Sá[1,2], Ana Rafaela Teixeira[1,2], Inês Loureiro[1,2,#], Catherine Thouvenot[3,#], Sylvain Golba [3], Rogerio Amino[4*] and Joana Tavares[1,2*]

[1] i3S – Instituto de Investigação e Inovação em Saúde, Universidade do Porto, Porto, 4200-135, Portugal.

[2] IBMC – Instituto de Biologia Molecular e Celular, Universidade do Porto, Porto, 4200-135, Portugal.

[3] Center for Production and Infection of Anopheles, Institut Pasteur, Paris, 75015, France.

[4] Unit of Malaria Infection and Immunity, Institut Pasteur, Paris, 75015, France.

# Current address: C. Thouvenot: Ultrapole, Institut Pasteur, Paris, 75015, France.

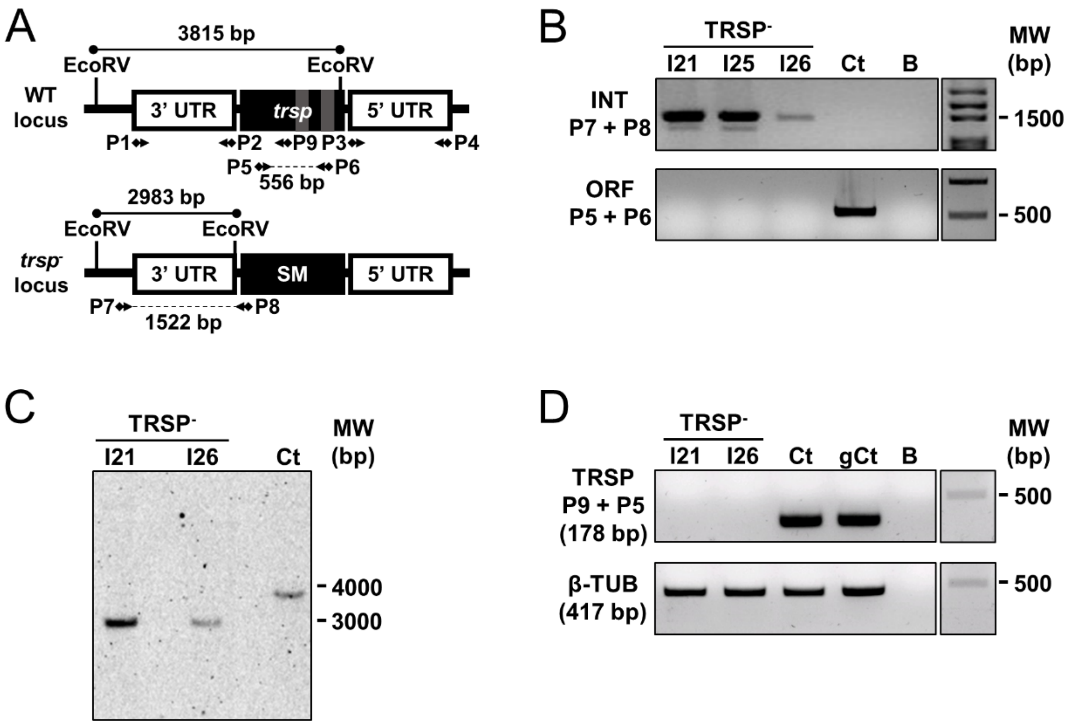

Supplementary Figure 1. Targeted gene replacement of *trsp* in GFP:LUC parasites. (A) Schematic representation of the *trsp* replacement strategy by homologous recombination in GFP:LUC parasites. The targeting vector used to generate the *trsp* knockout (*trsp*$^-$) locus was comprised of the selectable marker (SM), the *Toxoplasma gondii* dihydrofolate reductase-thymidylate synthase gene, flanked by 5' and 3' untranslated regions (UTRs) amplified with primers pairs P3 + P4 and P1 + P2 (Supplementary Table 1), respectively, which functioned as homology arms. Solid black or gray boxes in the wild type (WT) locus represent exons or introns, respectively. (B,C) Genetic analysis of TRSP$^-$ clones and control (Ct) parasites by PCR (B) and Southern blot (C). Primers used in the reactions, the restriction sites and the expected fragment (solid lines) or amplicon (dashed lines) length are specified in panel A. The sequence that served as the homology region downstream of the gene was used as the probe in C. (D) mRNA expression of TRSP in TRSP$^-$ I21 and I26 and control salivary gland sporozoites evaluated by RT-PCR. The predicted size of the amplicons is indicated to the left of the gels.

Genomic DNA extracted from control parasites (gCt) was used as a control PCR. The DNA ladder in B and D was run on the same gel as the PCR products on the left but the corresponding lanes were cropped from the respective images to facilitate visualization. B, blank (PCR without DNA). bp, base pairs. INT, integration PCR. ORF, open reading frame PCR. MW, molecular weight. TUB, tubulin beta chain.

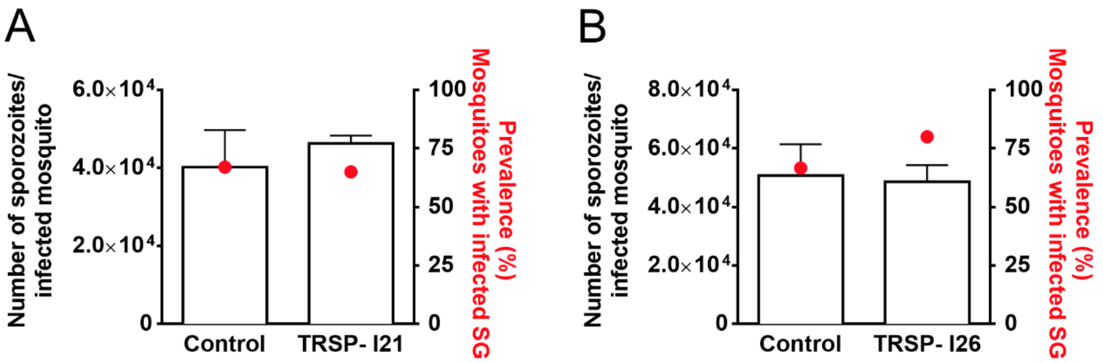

Supplementary Figure 2. Mosquito infectivity by TRSP$^-$ parasites. Percentage of mosquitos infected with control or either TRSP$^-$ I21 (A) or I26 (B) parasites determined by counting the number of GFP fluorescent salivary glands (SG) and total numbers of salivary gland sporozoites per infected mosquito (means + SD of two independent determinations). These data correspond to representative experiments.

Supplementary Table 1. Oligonucleotide sequences

Primer	Sequence
**1**	5’ TAATGGATCCGCAAAATATAGTTACACCCATA 3’
**2**	5’ TAATGAATTCGATAATAGTTTGGCTTTTCG 3’
**3**	5’ TAATAAGCTTCATATAAACTATGAACG 3’
**4**	5’ TAATGGTACCGTTTGTGCACCTTAATCCC 3’
**5**	5’ TCCCTTCAGAATTGTCAGGAC 3’
**6**	5’ GTGCTCAAATAATCAACCTGTTG 3’
**7**	5’ TGAGTGCACATAAGCAATTC 3’
**8**	5’ CCCATTGTGAACATCCTCAAC 3’
**9**	5’ AAACGAGTTGCGTAATTGTG 3’
**10**	5’ TGGAGCGGAAATAACTGGG 3’
**11**	5’ ACCTGACATAGCGGCTGAAA 3’

Introduced restriction sites are underlined.
